# The pandemic push: can COVID-19 reinvent conferences to models rooted in sustainability, equitability and inclusion?

**DOI:** 10.1007/s42532-020-00059-y

**Published:** 2020-08-25

**Authors:** Holly J. Niner, Shaili Johri, Judith Meyer, Sophia N. Wassermann

**Affiliations:** 1 School of Biological and Marine Sciences, Floor 3, Marine Building, University of Plymouth, Drake Circus, Plymouth, PL4 8AA UK; 2 Hopkins Marine Station, Department of Biology, Stanford University, 120 Oceanview Boulevard, Pacific Grove, CA 93950 USA; 3 Centre for Ocean and Society, Christian-Albrechts-University Kiel, Neufeldtstraße 10, 24118 Kiel, Germany; 4 Ryan Institute, School of Natural Sciences, National University of Ireland, Galway, University Rd., Galway, H91 TK33 Ireland

**Keywords:** Equitable access, Sustainability, Inclusion, Diversity, Knowledge exchange, Virtual conference, Online conference

## Abstract

The COVID-19 pandemic necessitates a change in conference formats for 2020. This shift offers a unique opportunity to address long-standing inequities in access and issues of sustainability associated with traditional conference formats, through testing online platforms. However, moving online is not a panacea for all of these concerns, particularly those arising from uneven distribution of access to the Internet and other technology. With conferences and events being forced to move online, this is a critical juncture to examine how online formats can be used to best effect and to reduce the inequities of in-person meetings. In this article, we highlight that a thoughtful and equitable move to online formats could vastly strengthen the global socio-ecological research community and foster cohesive and effective collaborations, with ecology and society being the ultimate beneficiaries.

## A window of opportunity

 International conferences are valued as important for the development of both researchers and knowledge (Fraser et al. 2017, p. 540; Timperley et al. 2020, pp. 11–12). Yet, the traditional conference model that brings delegates together in a single ‘destination’ demands costly international travel and often high registration fees. Given the associated carbon emissions and inequities in access, there is a particularly strong moral onus for those engaged in the field of socio-ecology to develop conference models or practices that do not contribute to the very problems that the discipline seeks to address.

The forcing hand of COVID-19 has opened an opportunity to trial online formats and to reinvent conferences as a core institution of research and practice. A global rise in community goodwill and flexibility in response to the challenges of ‘lockdown’ (Morgan 2020) provides an opportunity to address some of the long-term ethical quandaries that relate to both sustainability and accessibility (Ford et al. 2018; Arend and Bruijns 2019; Timperley et al. 2020) posed by traditional conference formats.

## Inequities of in-person conferences

Equal participation at conferences is restricted by access to financial support for travel and registration fees. The costs of visas, flights, registration and accommodation are an insurmountable obstacle for many involved in socio-ecological practice (Xiang 2019) and research. Funding for attendance is frequently limited for those in practitioner positions outside of the academy and those based in low-income nations (Fullick 2016; Waruru 2018). In our experience organising several non-profit international socio-ecological conferences, raising finance to support attendance in constrained funding landscapes remains challenging, despite the ethical and moral imperatives to diversify participation.

Beyond access, societies have moved to address issues of inequality faced by participants. These efforts include codes of conducts to manage in-conference interaction (Favaro et al. 2016), mentoring (Timperley et al. 2020, p. 12), childcare provision, employing mediators to manage disputes and incentivising leadership roles and participation of historically and structurally marginalised groups including women, people of colour, those with physical and mental health disabilities, and LGBTQ + (e.g. IMCC5 2017).

Additionally, travelling internationally to a conference is not sustainable. Some conferences offset their participants’ travel emissions (Holden et al. 2017, p. 1211). However, the compensation of carbon emissions is controversial and, according to the carbon management hierarchy, should only be used as a last resort after exhausting all other options for mitigation (Hyams and Fawcett 2013, p. 93).

## Inequities of online conferences

Online conference formats remove the need for travel and reduce the costs of attendance, but they do not preclude inequality in access and participation. New challenges are posed by a move online, such as replicating the much valued spontaneous and informal opportunities of traditional in-person conferences, where non-verbal cues are more easily detected (Fish et al. 1993, p. 46; Erickson 2011, p. 508). Furthermore, the different norms of interaction in an online setting may exacerbate inequalities in participation. For instance, online communication is often associated with a degradation in politeness (Hardaker 2010, p. 238). Cultural insensitivity and impoliteness are known causes of lower levels of minority faculty representation in the academy (Louque and Thompson 2005, p. 38 and p. 233). Accordingly, online communication may make it harder for inexperienced or minority community members to establish themselves in a global network of colleagues. This may have long-term impacts on the diversity and innovation potential of the socio-ecological research community.

Another immediate issue is access to technology and infrastructure for online participation. Minority participants likely experience the digital divide disproportionately. For example, only 42% of urban households in India have Internet access, dropping to 14.9% for rural households (Government of India 2018 p.47). Further, primary users of the Internet in India are male (36%) with only 16% of women having access to mobile Internet, the primary mode of digital connectivity (GSMA 2019, p. 15). This underlying disparity in digital access is partially neutralised by reliable Internet access for those engaged at a subset of governmental, private and higher education institutes. However, with the current shelter-in-place restrictions, institutional access is restricted, forcing users to rely on in-home or mobile Internet, which is frequently unavailable, with only 23.4% of urban households having access to a computer (Government of India 2018, p. 47). Access is likely even worse for conservation practitioners based in rural settings globally and for students ‘sent home’ from universities. In Africa in March 2020, only 39.3% of the total population could access the Internet, compared to the rest of the world at 62.9%, with smartphone access at 51% in South Africa, 30% in Kenya and extremely low at 13% in Tanzania (Ngware 2020).

Increasing global access to the Internet is central to achieving the UN sustainable development goals, and a shift to online conferences supports this aim. However, increasing access will increase the carbon emissions associated with the Internet, which are estimated to exceed those of the aviation industry (Boston Consulting Group 2012, pp. 9–13; Malmodin and Lundén 2018, pp. 26–29). Evidently, ‘going online’ does not completely neutralise the carbon emissions of a conference (Taylor 2020). As such, accounting for the carbon footprint of conferences remains relevant for online formats, but could legitimately meet the demands of the carbon management hierarchy.

## Supporting an equitable online future

The COVID-19 pandemic offers an opportunity to understand and demonstrate how online platforms can address issues of equity in access and sustainability. The elimination of travel costs clearly reduces the barriers to participation as long as conference registration fees associated with in-person events are similarly reduced. However, engagement online must be fostered to allow online formats to confer the same value as traditional formats. Additionally, the associated technological requirements could risk widening the inequities for participants who are already the most disadvantaged in the socio-ecological community (Martin 2012; UN News 2020).

A key ‘entry cost’ to participation remains in the issue of technology access and Internet infrastructure. While many conference participants may have adequate access to Internet and technology, to address issues of diversity, equity and inclusion, online platform selection should consider associated requirements for high bandwidth, high-performing devices and training in these technologies. In the absence of uniform digital access, conferences can make use of a variety of more widely available technologies, such as dissemination of conference content in recorded, live and audio-only formats accessible via telephone, radio (Ngware 2020), podcasts and other popular smartphone applications such as WhatsApp (Bouhnik and Deshen 2014, pp. 217–220; Taru 2020).

Meeting format can address inequalities in participation, but success will depend on community goodwill to actively engage in the programme. Formats can be adapted to support the cultivation of ‘social presence’ through careful consideration of scheduling around time zones, and a combination of live and recorded presentations and interactive events, such as live question and answer sessions or break out groups (Tu and McIsaac 2010). Mentorship and clear communication of expectations of engagement as set out by a code of conduct could also assist in supporting aims of equitable inclusion and in providing space for all voices to be heard. The optimal model for online conferences may differ more drastically from the in-person format. Rather than large, immersive conferences held over a set time period, it could include perennial platforms (e.g. professional societies or established communication hubs) integrating a range of technologies that host ‘special issues’ or informal networking events. These ‘smaller’ yet more frequent events may ensure active and sustained participation across geographic and disciplinary sectors, ensuring a true diverse and inclusive conference model.

## Conclusions

The potential to address long-standing inequities in the socio-ecological community through online conferences is a bright spot in the post-COVID-19 landscape. For now, online formats tend to follow the traditions of in-person conferences, being focussed over a defined period and based around thematically grouped presentations or posters. The current pandemic has led to a shift in forms of communication (Taru 2020; Wen 2020), and as people adapt to forging and maintaining relationships online rather than in person, innovation of the conference model to avoid the risks of continuing or exacerbating issues of inclusion and access online will be key.

The reinvention of conferences required by COVID-19 shows us that there are viable options for professional and knowledge development that do not sit at odds with ambitions for an equitable and sustainable future. However, this reinvention must carefully consider the requirements for equitable access and will depend on ongoing and enthusiastic engagement of audiences. As the COVID-19 response challenges the need for and moral standing of the traditional conference, we have been given an opportunity to experiment and begin to explore what works best for all sectors of society.
